# Supplementary material for: Big Data to Knowledge Analytics Reveals the Zika Virus Epidemic as Only One of Multiple Factors Contributing to a Year-Over-Year 28-Fold Increase in Microcephaly Incidence
Source: Int J Environ Res Public Health. 2022 Jul 25;19(15):9051. doi: 10.3390/ijerph19159051 (PMC9331749; doi:10.3390/ijerph19159051)
Supplement: Supplementary file 1 [file ijerph-19-09051-s001.zip › ijerph-1731959-supplementary.pdf]

Supplementary Material

BD2K analytics reveals the Zika virus epidemic as only one of multiple factors contributing to year-over-year 28-fold increase in microcephaly incidence

Myriam Patricia Cifuentes,[1,2*] Clara Mercedes Suarez,[3] Ricardo Cifuentes,[4] Noel Malod-Dognin,[5] Sam Windels,[5] Jose Fernando Valderrama,[6] Paul D. Juarez,[7] R. Burciaga Valdez,[8] Cynthia Colen,[9] Charles Phillips,[13] Aramandla Ramesh,[10] Wansoo Im,[7] Maureen Lichtveld,[14] Charles Mouton,[11] Natasa Przulj,[5] and Darryl B. Hood[2*]

This file includes:

Materials and Methods
Figures S1, S2 and S3
Tables S1 and S2

* Corresponding author; Darryl B. Hood, Ph.D., Professor, Division of Environmental Health Sciences, The Ohio State University; Email, hood.188@osu.edu

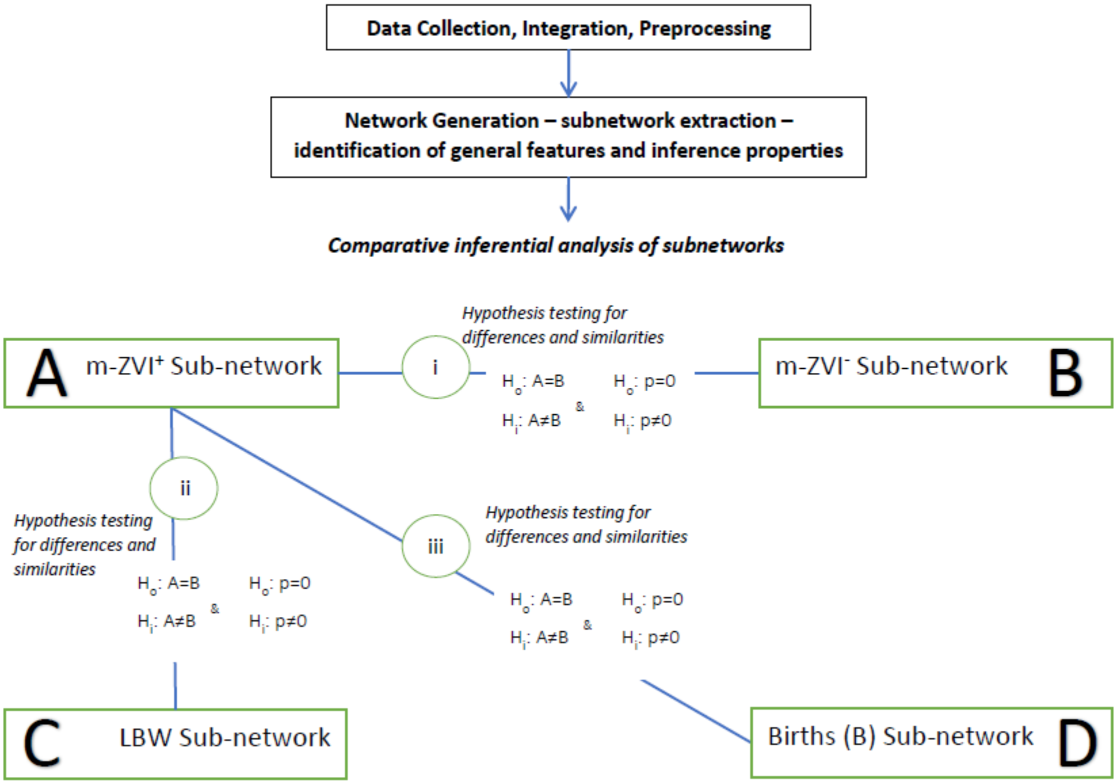

Figure S1. Methodology Flowchart. Subsequent to data collection, integration, preprocessing and network generation, the third step of model analysis consisted of extraction and comparison of 4-subnetworks to test three morbidity hypotheses. Subnetworks are identified by letters and comparisons between networks by lower-case roman numerals.

Materials and Methods

We generated a complex network of interdependent determinants of ZVI-microcephaly by inferring associations between variables describing diverse factors and the incidence of microcephaly attributed to ZVI in Brazil municipalities during the first semester of 2016. This network provided the context to extract sub-networks of factors associated to ZVI^{+}-microcephaly and controls that allowed to perform comparative inferential analysis beyond a descriptive approach.

Figure S2. Threshold Criteria for variable selection

Threshold of the distribution of Pearson coefficients according to literature (0.7)

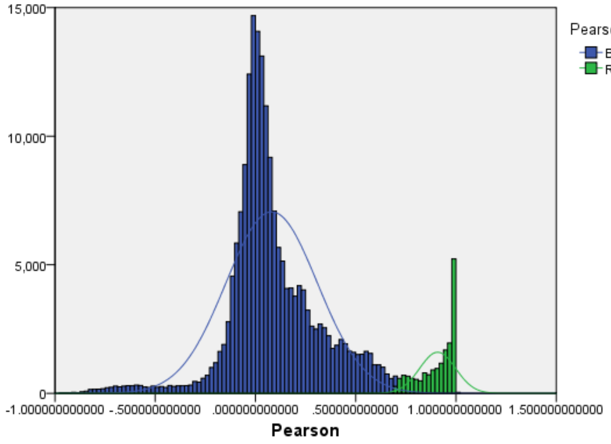

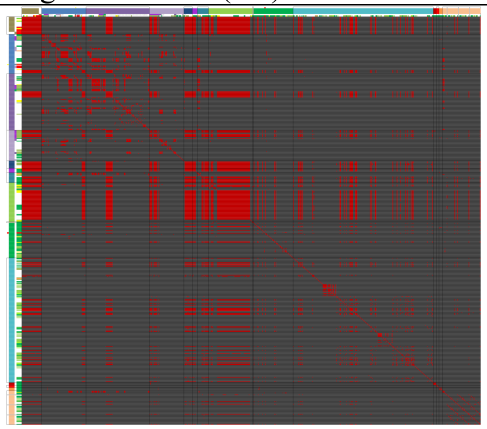

Red: >0.7
Black: <=0.7

Thresholds based on 2.5 standard deviations of the distribution of Pearson coefficients

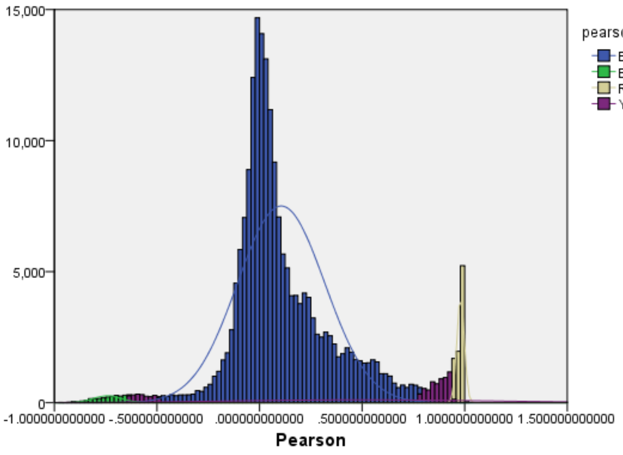

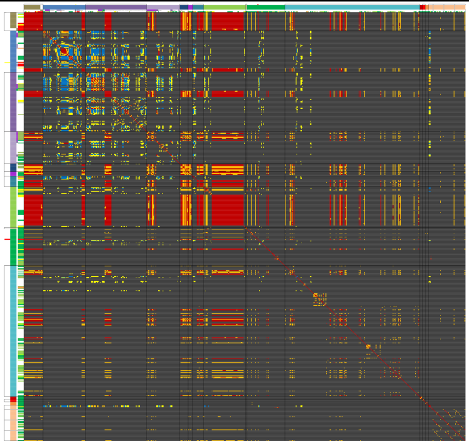

Red: (0.987, 1]
Blue: [1, -0.749)
Yellow [-0.749, 0.605) & (0.842-0.987]
Black: [-0.605, 0.842]

Thresholds based on 2.5 IQR of the distribution of Pearson coefficients

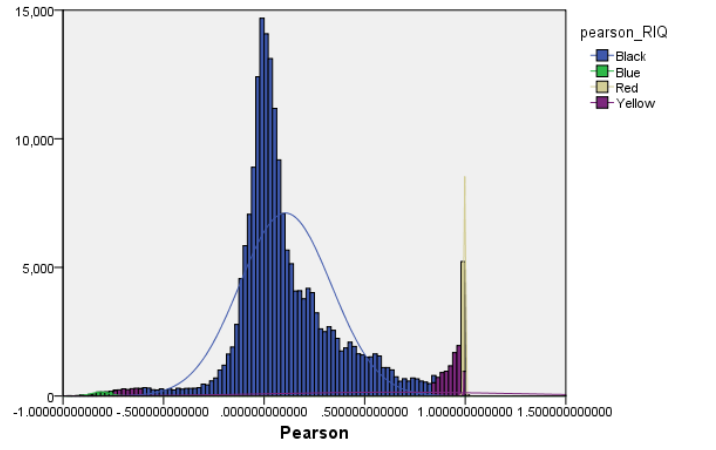

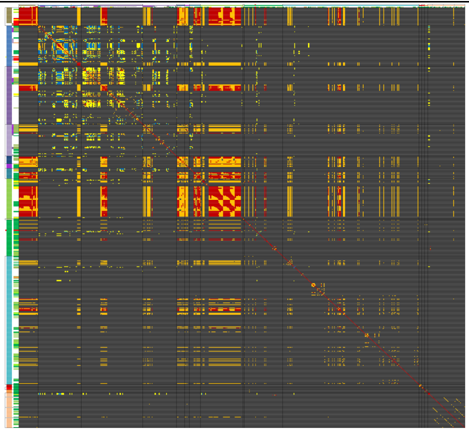

Red (0.987,1]
Blue [-1, -0.749)
Yellow (-0.605, -0.749] & [0.842, 0.987]
Black [-0.605-0.842]

Thresholds based on 2 (left) nd 3 (right) Standard deviations of Pearson Fisher-z transform

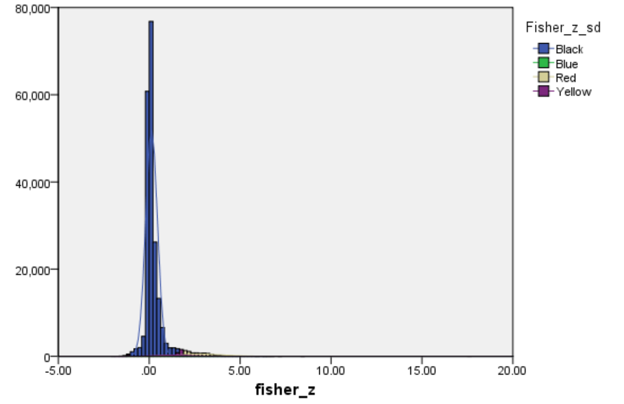

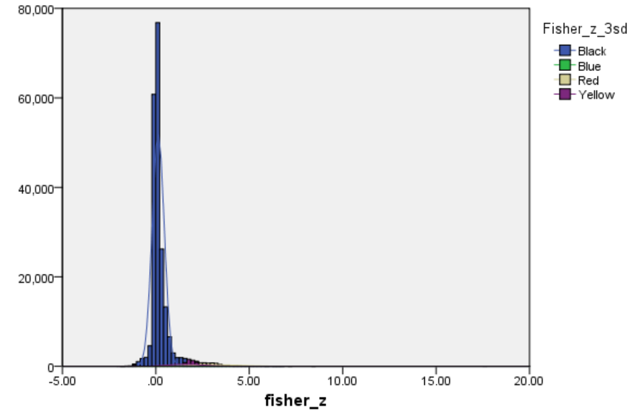

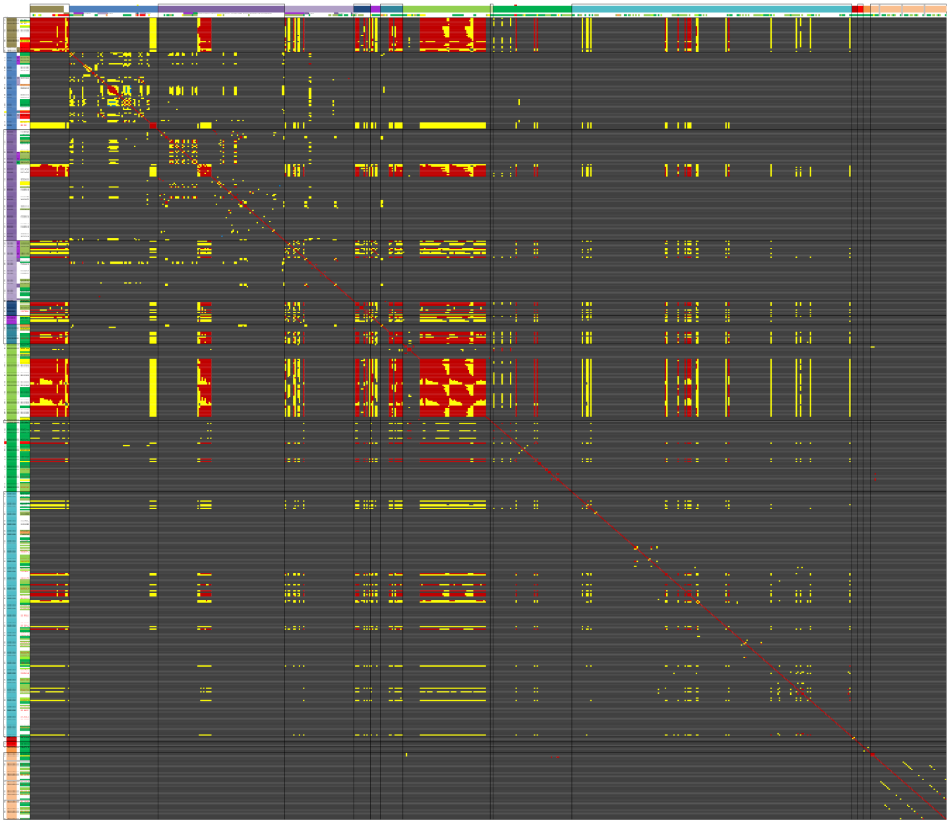

Red (0.977, 1]
Blue [-1, -0.946)
Yellow [-0.946, -0.797) & (0.917, 0.977]
Black [-0.797, 0.917]

Thresholds based on 2.5 (left) and 3 (right) IQR of Pearson Fisher-z transform

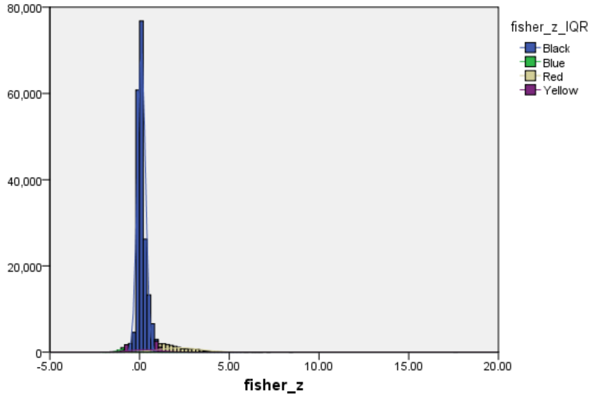

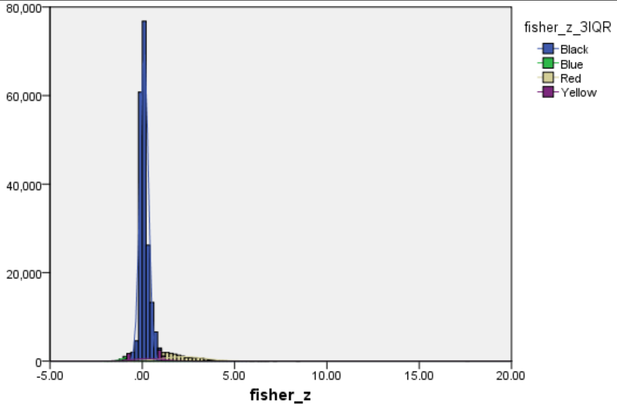

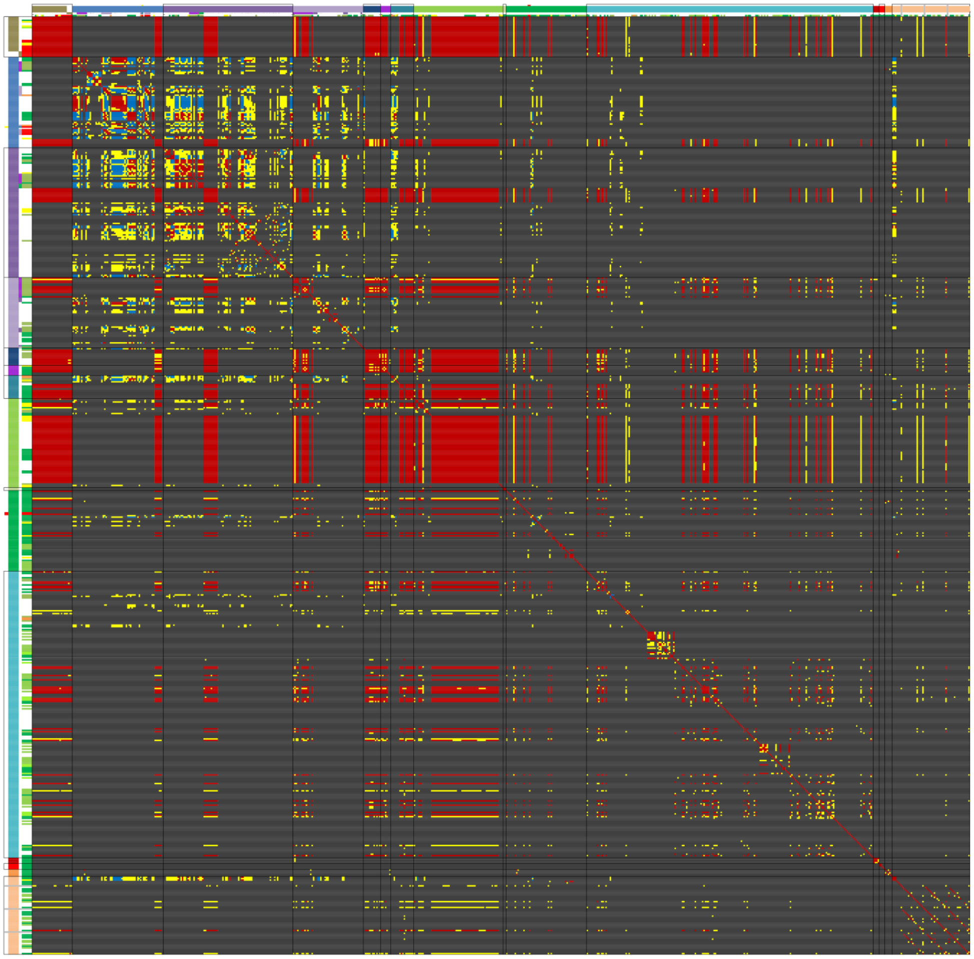

Red (0.820, 1]
Blue [-1, -0.968)
Yellow [-0.968, -0.549) & (0.697, 0.820]
Black [-0.549, 0.697]

Thresholds based on the distribution of VIF according to literature (>10)

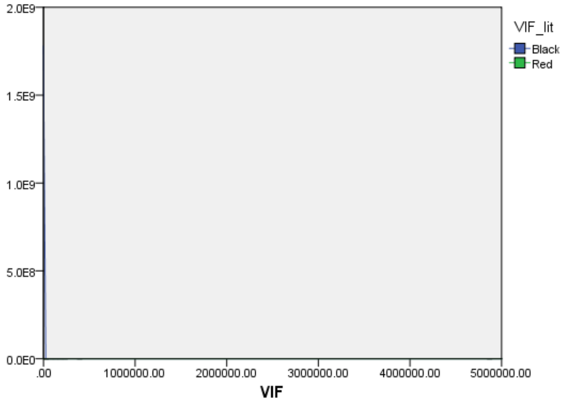

Thresholds based on 2.5 Standard deviations of VIF distribution

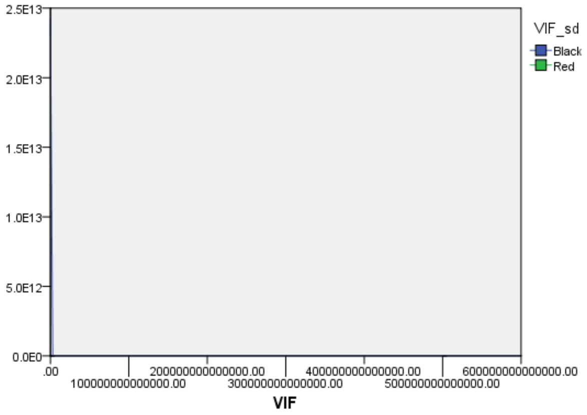

Thresholds based on 3 Standard deviations of VIF distribution

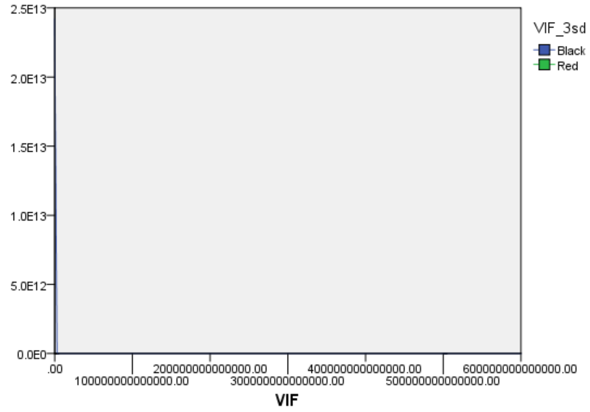

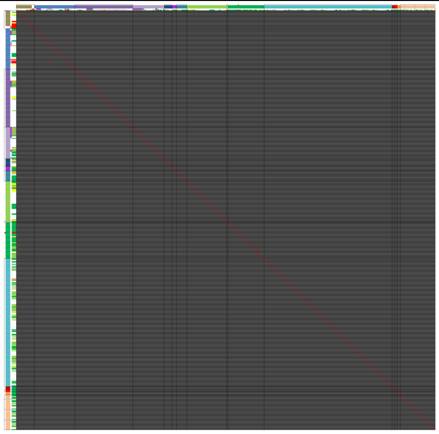

Red [-1, -0.968) & (0.999, 1]
Black [-0.968 & 0.999]

Thresholds based on 2.5 (left) and 3 (right) IQR of VIF distribution

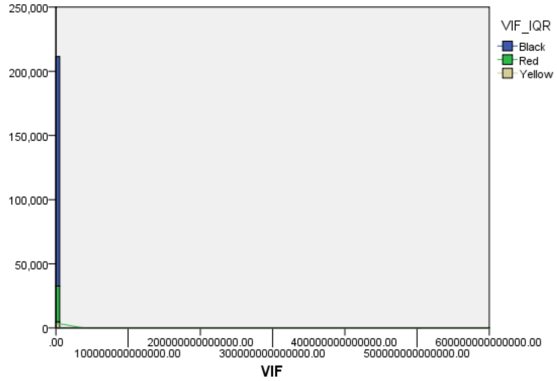

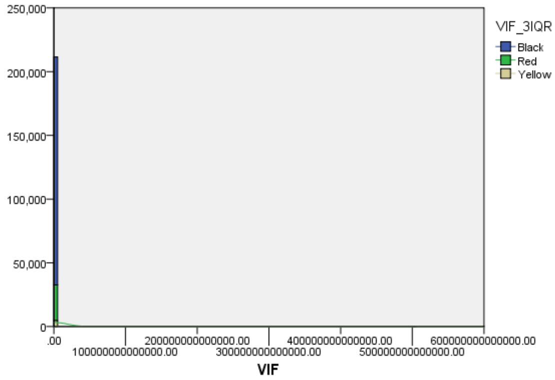

Matrix highlighting variables by groups of determinants according to the thresholds

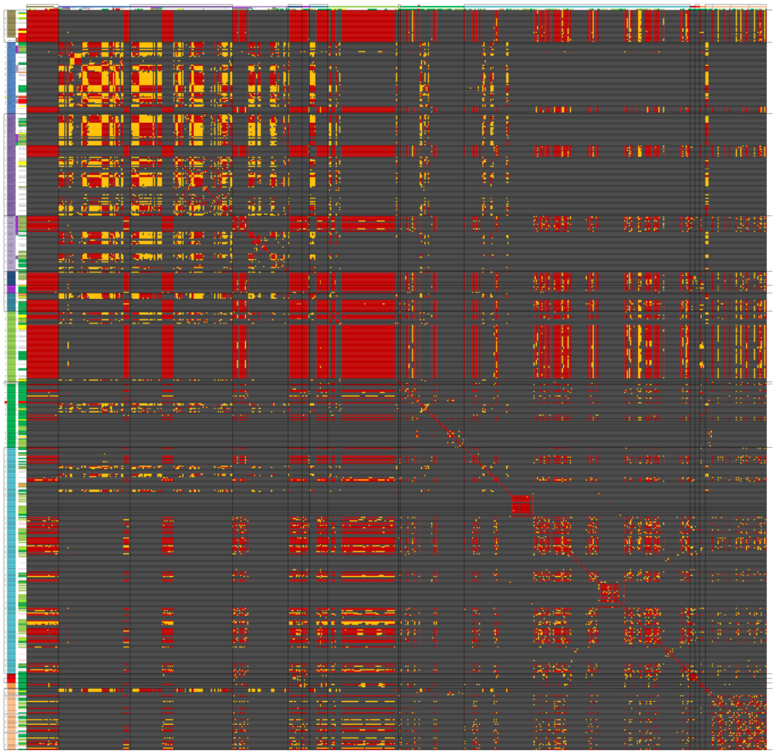

Red [-1, -0.968) & (0.552-1]
Yellow [-0.968, -0.498) & (0.498, 0.552]
Black [-0.498, 0.498]

<table>
<tr><td colspan="2">Thresholds based on 2 and 2.5 standard deviations of the distribution of Spearman coefficients</td></tr>
<tr><td>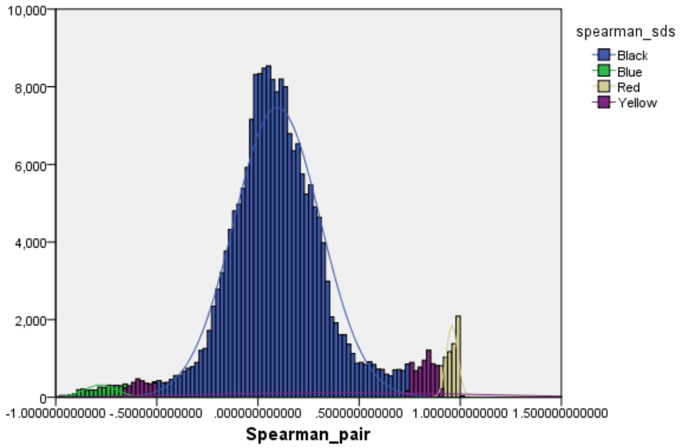
</td><td>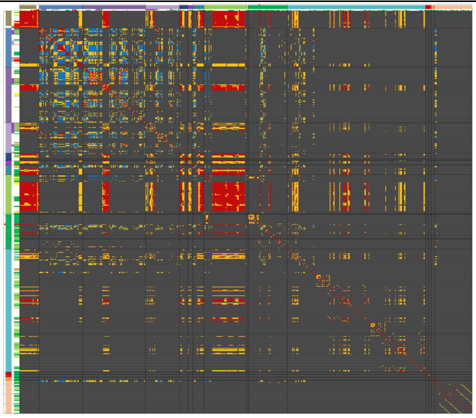
Red (0.902, 1]
Blue [-1, -0.658)
Yellow [-0.658, -0.502) & (0.746, 0.902]
Black [-0.502, 0.746]</td></tr>
<tr><td colspan="2">Thresholds based on 2 and 2.5 IQR of the distribution of Spearman coefficients</td></tr>
<tr><td>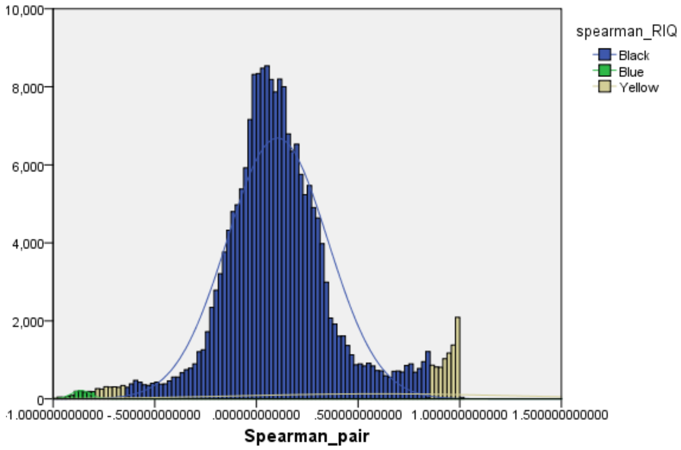
</td><td>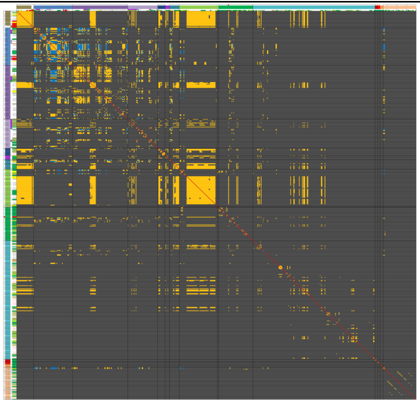
Red (1, 1]
Blue [-1, -0.792)
Yellow [-0.792, -0.642) & (0.854, 1]
Black [-0.642, 0.854]</td></tr>
</table>

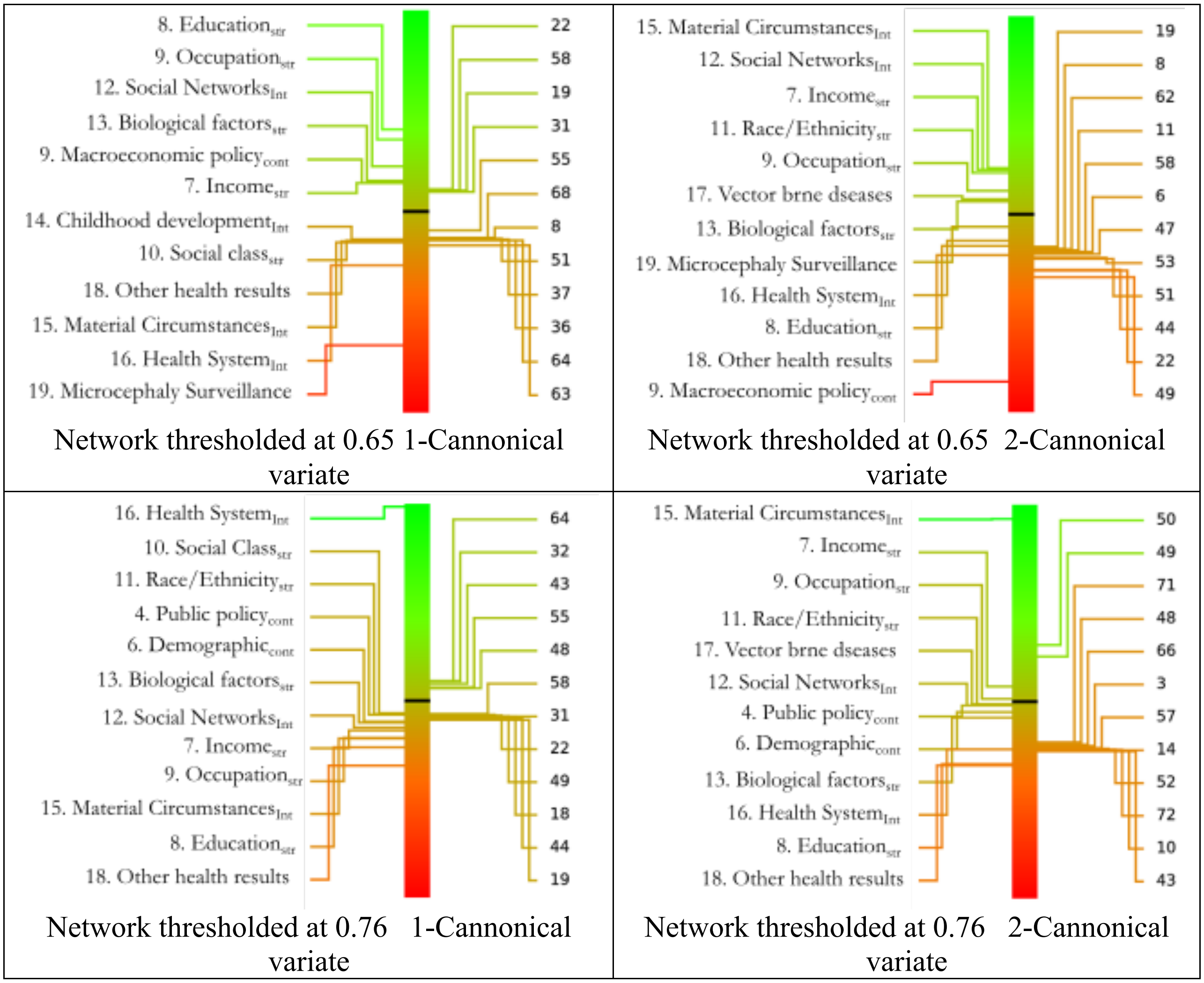

Figure S3. Red-green plots of Canonical Analysis loadings for the relationships at the local structure (orbit level) and the determinant factors.

Table S1. Global indices of the general and thresholded networks

Indices/Network		General network	Thresholded pcor >|0.65| network	Thresholded pcor >|0.76| network
Centrality Indices	Nodes	381	381	377
	Links	36,400	27,323	21,753
	Density	0.50	0.37	0.30
	Avg. Distance	1.51 (Max 4)	1.7 (max 4)	2.07 (max 5)
	Avg. Degree	190.57	143.05	113.89
	Degree Centralization	0.26 (99.9% CI* [0.05-0.11])	0.21	0.16
	Avg. Closeness	0.66	0.59	0.48
	Closeness Centralization	n.a.	n.a.	n.a.
	Avg. Betweenness	0.0013	0.0018	0.0027
	Betweenness Centralization	0.0070 (99.9% CI* [0.0003-0.0007])	0.014	0.029
Watts –Strogatz Clustering Coefficient		0.77 (99.9% CI* [0.49-0.50])	0.79	0.79
Clustering Coefficient 1		0.78 (99.9% CI* [0.49-0.50])	0.83	0.86
Undirected triad census	Empty Triads	925,158 (99.9% CI* [1,106,663-1,193,165])	2,067,972	3,149,964
	One Edge Triads	4,702,422 (99.9% CI* [3,409,366-3,495,019])	5,154,589	4,753,097
	Intransitive Triads	1,640,662 (99.9% CI* [3,416,707-3,503,300])	757,146	430,754
	Transitive Triads	1,949,418 (99.9% CI* [1,113,728-1,198,329])	1,237,953	883,845
	Ratio intrans/trans	0.84	0.61	0.49

*Confidence intervals of each index in the sampling distribution by MCS. Model's index values out of the interval confidence indicate significant differences of the network to ER model.

'Closeness' and 'closeness centralization' enclosed distribution of proximity among nodes; 'betweenness' and 'betweenness centrality' refer to distribution of mediation; Watts Strogatz and clustering coefficients (CC1 and CC2) addressed general and each node's ability to form groups with 1 and 2 direct neighbors (*19*).

Table S2. Subnetwork Indices

Subnetwork		m-ZVI+					m-ZVI-					LBW					Births				
Different thresholds		Full	TR65	Δ	TR76	Δ	Full	TR65	Δ	TR76	Δ	Full	TR 65	Δ	TR 76	Δ	Full	TR 65	Δ	TR 76	Δ
Centrality Indices	Nodes	171	144	0.84	118	0.69	174	140	0.80	118	0.68	247	129	0.52	92	0.37	262	142	0.54	57	0.22
	Links	11779	7863	0.67	5131	0.44	12007	7,637	0.64	5,153	0.43	17,276	5,708	0.33	3,099	0.18	19,449	5,465	0.28	1,030	0.05
	Density	0.81	0.76	0.94	0.7433	0.92	0.798	0.78	0.98	0.75	0.94	0.569	0.69	1.21	0.74	1.30	0.569	0.54	0.95	0.64	1.12
	Avg distance	1.18961	1.24	1.04	1.26	1.06	1.20225	1.21	1.01	1.25	1.04	1.43136	1.31	0.92	1.26	0.88	1.43117	1.45	1.01	1.35	0.94
	Avg. Degree	137.766	109.21	0.79	86.97	0.63	138.011	109.1	0.79	87.34	0.63	139.887	88.5	0.63	67.37	0.48	148.466	76.98	0.52	36.14	0.24
	Degree centralization	0.19	0.24	1.25	0.26	1.35	0.20	0.22	1.08	0.26	1.27	0.44	0.31	0.71	0.26	0.60	0.43	0.46	1.06	0.37	0.85
	Avg. Closeness	0.85	0.82	0.96	0.81	0.95	0.84	0.84	0.99	0.81	0.96	0.71	0.78	1.11	0.81	1.15	0.71	0.69	0.98	0.75	1.06
	Closeness centralization	0.30	0.36	1.22	0.38	1.28	0.31	0.33	1.05	0.38	1.21	0.59	0.45	0.76	0.39	0.66	0.59	0.62	1.05	0.51	0.86
	Avg. betweenness	0.0011	0.0016	1.45	0.0022	2.00	0.0012	0.0015	1.25	0.0022	1.83	0.0018	0.0024	1.36	0.0028	1.59	0.0017	0.0032	1.93	0.0064	3.86
	Betweenness centralization	0.0016	0.0040	2.50	0.0047	2.94	0.0018	0.0029	1.61	0.0043	2.39	0.0187	0.0143	0.76	0.0120	0.64	0.0226	0.0557	2.46	0.0390	1.73
Watts –Strogatz clustering coefficient		0.89	0.87	0.98	0.86	0.97	0.88	0.88	1.00	0.86	0.98	0.86	0.87	1.01	0.88	1.02	0.91	0.87	0.96	0.85	0.98
Clustering coefficient 1		0.87	0.85	0.97	0.83	0.95	0.87	0.86	0.99	0.83	0.96	0.81	0.85	1.05	0.86	1.06	0.87	0.84	0.96	0.82	0.98
Undirected triad census	Empty triads (1-003)	10,507	9,415	0.90	7,209	0.69	12,701	7,610	0.60	7,041	0.55	92,939	8,033	0.09	2,955	0.03	51,208	16,709	0.33	965	0.02
	One edge triads (3-102)	110,358	90,588	0.82	52,914	0.48	127,911	71,670	0.56	51,728	0.40	1,227,089	111,079	0.09	29,757	0.02	1,645,334	257,327	0.16	10,587	0.01
	Intransitive triads (11-201)	213,527	136,065	0.64	78,097	0.37	229,643	122,664	0.53	78,421	0.34	477,730	77,339	0.16	29,451	0.06	388,628	71,519	0.18	7,057	0.02
	Transitive triads (16-300)	484,413	251,276	0.52	128,696	0.27	492,669	245,636	0.50	129,726	0.26	683,357	153,053	0.22	63,417	0.09	878,050	121,625	0.14	10,649	0.01
	ratio intrans trans	0.44	0.54	1.23	0.61	1.38	0.47	0.50	1.07	0.60	1.30	0.70	0.51	0.72	0.46	0.66	0.44	0.59	1.33	0.66	1.50

Table includes network indices of full versions of subnetworks (defined according to Hartemink's mutual information based-thresholds of pcor) and further thresholded according to structural criteria by Graphlet distributions and Clustering Coefficients. Columns marked with Δ present the proportion that remains in each resulting index (preceding column) after thresholding by comparing its value with the equivalent one in the full network. To improve visualization, we added heat maps to Δ columns: green shades mark changes above unit (increase), red shades mark changes below the unit (decrease) and white cells mark values around 1.0
